# Impact of opioid-free analgesia on pain severity and patient satisfaction after discharge from surgery: multispecialty, prospective cohort study in 25 countries

**DOI:** 10.1093/bjs/znad421

**Published:** 2024-01-11

**Authors:** TASMAN Collaborative, William Xu, Gordon Liu, Chris Varghese, Cameron Wells, Nicolas Smith, John Windsor, Lorane Gaborit, Sarah Goh, Aya Basam, Muhammed Elhadi, Rachel TingQian Soh, Umar Saeed, Eman Abdulwahed, Michael Farrell, Deborah Wright, Jennifer Martin, Peter Pockney, William Xu, Aya Basam, Sarah Goh, Jiting Li, Jainil Shah, Abdullah Waraich, Lorane Gaborit, Upasana Pathak, Amie Hilder, Muhammed Elhadi, Aiden Jabur, Kaviya Kalyanasundaram, Christina Ohis, Chui Foong Ong, Melissa Park, Venesa Siribaddana, Kyle Raubenheimer, Jennifer Vu, Cameron Wells, Gordon Liu, Liam Ferguson, William Xu, Chris Varghese, Peter Pockney, Kristy Atherton, Amanda Dawson, Jennifer Martin, Arnab Banerjee, Nagendra Dudi-Venkata, Nicholas Lightfoot, Isabella Ludbrook, Luke Peters, Rachel Sara, David Watson, Deborah Wright, Ademola Adeyeye, Luis Adrian Alvarez-Lozada, Semra Demirli Atici, Milos Buhavac, Giacomo Calini, Muhammed Elhadi, Orestis Ioannidis, Mustafa Deniz Tepe, Upanmanyu Nath, Ahmad Uzair, Wah Yang, Faseeh Zaidi, Surya Singh, Bahiyah Abdullah, Diana Sofia Garces Palacios, Ahmed Ragab, Ahmed Ahmed, Kyle Raubenheimer, Davina Daudu, Sarah Goh, Simran Vinod Benyani, Nandini Karthikeyan, Laure Taher Mansour, Warren Seow, Zoya Tasi, Aiden Jabur, Upasana Pathak, Melissa Park, Dhia Errahmane Abdelmelek, Ikram Fatima Zohra Boussahel, Oumelaz Kaabache, Naoual Lemdaoui, Oualid Nebbar, Mounira Rais, Meriem Abdoun, Aya Tinhinane Kouicem, Souad Bouaoud, Kamel Bouchenak, Hind Saada, Amel Ouyahia, Wassila Messai, Zhi Shyuan Choong, Clarissa Ting, Michelle Larkin, Pei Jun Fong, Isabel Soh, Alyssia De Grandi, Hareem Iftikhar, Akansha Sinha, Dhruv Kapoor, Tara Chlebicka, David Singer, Kim Goddard, Lisa Matthews, Rosalina Lin, Jessica Chambers, Juliet Chan, Brooke Macnab, John Barker, Morgan Mckenzie, Neil Ferguson, Ghanisht Juwaheer, Vijayaragavan Muralidharan, Sonia Gill, Nakjun Sung, Rohan Patel, Chris Walters, Kevin Nguyen, David Liu, Carlos Cabalag, Jennifer Lee, San-Hui Anita Leow, Suat Li Ng, Hamza Ashraf, Fraizer Mulder, Jonathan Loo, David Proud, Samantha Wong, Yida Zhou, Qi Rui Soh, David Chye, Sean Stevens, Patrick Tang, Stephen Kritharides, Jason Dong, Oscar Morice, Dora Huang, Andrew Hardidge, Mishka Amarasekara, Aleah Kink, Damien Bolton, Alisha Rawal, Jasraaj Singh, Matthew Heard, Yusuf Hassan, Ahmed Naqeeb, Andrew Cobden, Duron Prinsloo, Dwain Quadros, Emma Gunn, Ha Jin Kim, Jennifer Ekwebelam, James Shanahan, Mustafa Alkazali, Mariyah Hoosenally, Naveen Nara, Peter Nguyen, Sally Barker, Zacchary Tamsett, Naomi Rigby, Hinal Patel, Eloise Ferguson, Lauren Byrnes, Alexander Bravo, Amie Hilder, Ally Hui, Antara Karmakar, Bill Wang, Janindu Goonawardena, King Tung Cheung, Nicholas Chan, Ragul Natarajan, Richard Cade, Rong Jin, Shomik Sengupta, Ruth Snider, Harsha Morisetty, Lewis Weeda, Phoebe Sun, Lalitya Chilaka, Jacinta Cover, Aashrinee De Silva Abeweera Gunasekara, Rahavi Senthilrajan, Anas Alwahaib, Alexandra Limmer, Bushra Zamanbandhon, Kumail Jaffry, Yijia Shen, Alan Chua, Saifulla Syed, Sushanth Saha, John Glynatsis, Lori Aitchison, Bernard Lagana, Mason Crossman, David Watson, Abby Dawson, Bryan Fong, Ella Harrison, Eleanor Horsburgh, John Glynatsis, Michael Khoo, Kritika Mishra, Lewis Hewton, Alex Mesecke, Hien Tu, Than Tun, Jason Wong, Elynn Ong, Tara-Nyssa Law, Ashlee Landy, Alyssa Leano, Andrea Li, Akshay Soni, Benjamin Dowdle, Charles Pilgrim, Dewmi Abeysirigunawardana, Deepak Rajan Jeyarajan, Diya Patel, Jason Chung, Kyle Mckinnon, Madeline Gould, Paul Gilmore, Ruxi Geng, Rachael Loughnan, Sarahjane Norton-Smith, Solomon Nyame, Sarah Tan, Sewni Samarawickrama, Si Woo Yoon, Yantong Wang, Yichi Zhang, Zixuan Wang, Hans Mare, Indrajith Withanage, Mitali Khattar, Alexandra Toft, Goutham Sivasuthan, Hailin Zhao, Jordan Addley, Lucinda O'brien, Muhammad Raza, Randipsingh Bindra, Sonakshi Sharma, Charlotte Cornwell, Aditya Patil, Aiden Cheung, Ashleigh Lown, Amanda Dawson, Aneel Blassey, Benjamin Ochigbo, Felicity Cheng, Aleeza Fatima, Edward Zhang, Henry Kocatekin, Charles Roth, Dani Brewster, Kelvin Kwok, Paul Chen, Sharon Laura, Dominic Tynan, Edward Latif, Elizabeth Lun, Elodie Honore, Felix Ziergiebel, Jessica Blake, Karan Chandiok, Katie Bird, Lynette Ngothanh, Melissa Lee, Mariam El-Masry, Peter Hamer, Ramanathan Rm Palaniappan, Richard Mcgee, Sarah Huang, Shane Zhang, Shubhang Hariharan, Yannick De Silva, Celeste Lee, Penelope Fotheringham, Ian Incoll, Timothy Cordingley, Felicity Cheng, Matthew Brown, Leannedra Kang, Rivindu Wijayaratne, Parisse Moore, Gemma Qian, Yara Elgindy, Emma Carnuccio, Hamish Rae, Mena Shehata, Mingchun Liu, Brodee Lockwood, John Van Bockxmeer, Ali Alsoudani, Daniel Swan, Justin Hsieh, Francesca Orchard-Hall, Kai Yun Jodene Tay, Raagini Mehra, Alpha Gebeh, Ashley Bailey, Georgia Brown, Ashley Colaco, Hemashree Gopal, Jessica Boyley, Varun Changati, Joseph Fletcher, Tanishq Khandelwal, Colin House, Chris O'neil, Emily Jaarsma, Victor Ly, Zsolt Balogh, Amanda Shui, Vinogi Sathasivam, Hannah Legge-Wilkinson, King Ho Wong, Andrew Chen, Anthony Tran, Peter Rehfisch, Grace Wang, Jonathan Nguyen, Joshua Peker, Kayla Gallert, Mia Komesaroff, Manideep Namburi, Elisabeth Goldfinch, Ropafadzo Muchabaiwa, Aishwarya Jangam, Isobel Taylor, Iulian Nusem, Jin Hyuk (David) Park, Justin Gundara, Rachael Heigan, Tam Tran, Thomas Mackay, Yasmine Butterworth, Tomas Sadauskas, Melody Tung, Hasthika Ellepola, Christine Gan, Hakim Fong, Ankita Das, Leshya Naicker, Samantha Hauptman, Aditi Kamath, Anthea Yew, Anupam Parange, Katie Kim, Sahil Kharwadkar, Tharushi Gamage, Lucille Vance, Alexandra Seldon, Moheb Ghaly, Jainam Shah, Victoria Phan, Karanjeet Chauhan, Ahmad Bassam, Beverley Vollenhoven, Kumail Jaffry, Kajal Mandhan, Mithra Sritharan, Mahesh Sakthivel, Natalie Evans, Samuel Robinson, Seiyon Sivakumar, Liberty Marrison, David Jollow, Krishma Joshi, Steve Tao, Pallavi Shrestha, Sai Keerthana Nukala, Russell Hodgson, Anna Crotty, Adriana Esho, Alasdair Harris, Amy Surkitt, Laura Bland, Blake Mcleod, Chonghao Yin, Cambo Keng, Emily Greenwood, Grace Yuan, Emma Haege, Hongyi Wu, Haotian Xiao, Isabella Pozzi, Jeff Fu, Jessica Stott Ross, Juliette Gentle, Kathy Gan, Kelvin Chang, Kexin Sun, Madhavi Singh, Maria Xie, Nicholas Mccabe, Mark Slavec, Nick Clarnette, Behzad Niknami, Peishan Zou, Sean Flintoft, Shenuka Jayatilleke, Rumnea Sok, Suqi Tan, Sanya Wadhwa, Will Swansson, Daniel Abulafia, Jian Blundell, Amie Sweetapple, Caitlin Del Solar, Cameron Martin, David Bell, Isuru Fernando, Jared Chang, Katie Vanzuylekom, Katie Van Zuylekom, Kate Van Zuylekom, Katie Hobbs, Richard Liang, Aiden Jabur, Jazmina Tarmidi, Mahmoud Ugool, Nicholas Beatson, Sarah Bowman, Sophie Moin, Wen Po Jonathan Tan, Seevakan Chidambaram, Siang Wei Gan, Pengnan Wang, Leshya Naicker, Katie Kim, Nicole Qiwen Wang, Yi Xin Kwan, Chinmai Patil, Divyanshu Joshi, Aditi Kamath, Aishath Hanan, Arfaan Sheriff, Jaime Duffield, Leshya Naiker, Peter Smitham, Eu Ling Neo, Matthew Chua, Shalvin Prasad, Armitesh Nagaratnam, Tarik Sammour, Yuxin Lin, Christine Lee, Eve Hopping, Muskan Jangra, Ankita Das, Ken Lin, Zachary Bunjo, Kyle Raubenheimer, Mohamed Haseef Mohamed Yunos, Kar Long Yeung, Rachel Phu, Aisling Betts, Benjamin Just, Sahil Gera, Hilary Leeson, Jodie Jamieson, Katie Wang, Emily Luu, Michael Innes, Jennifer Vu, Jonathan Hong, Stephen Dzator, Aki Flame, Vincent Jiang, Jianing Kwok, Aaron Lawrence, Kate Meads, Liam Pearce, Pavatharane Sarangadasa, Haylee Shaw, Victor Yu, Elizabeth Crostella, James Wong, Sriya Bobba, Maddison Muller, Yin Chi Hebe Hau, Thomas Wilson, Aleksandra Markovic, Jemma Green, Clara Forbes, Emalee Burrows, Lachlan Hou, Clare O'sullivan, Jonathon Foo, Hannah Greig, A-J Collins, Callum Chandler, Emily Heaney, Hannah Gross, Monica Morgan, Rebecca Loder, Krishnankutty Rajesh, Shravankrishna Ananthapadmanabhan, Akeedh Razmi, Crystal Vong, Prasanna Pothukuchi, Mary Theophilus, Roshni Sriranjan, Sharon Kaur, Marcelo Kanczuk, Julia De Groot, Angela Corrigan, Damon Li, Danniel Badri, Dominico Ciranni, Elangovan Thaya Needi, Matthew Clanfield, Nicolas Copertino, William Rumble, Maria Kristina Vanguardia, Chen Lew, Rami Dennaoui, Jainil Shah, Joseph Kong, Imogen Koh, Raymond Zeng, Kristian Baziotis-Kalfas, Hannah Denby, Andy Li, Will Tran, Abhinav Singh, Olivia Lin, Michelle Chau, Olivia Donaldson, Christina (Seojung) Min, Shirahn Ballah, Sonia Ching Ting Tsui, Nathania Yong, Lucy Standish, Sarah Tan, Asuka Fujihara, Lily Davies, Ramin Odisho, Anjana Ravi, Josh Collins, Pooja Chandra, Rana Abdelmeguid, Gopal Singh, Xireaili Feierdaiweisi, Dharani Seneviratne, Shambhavi Srivastava, Michelle Yao, Cherilyn Teng, Nebula Chowdhury, Sasini Vidanagama, Charles Lin, Tharushi Sampatha-Waduge, Erica Wang, Chatnapa Yodkitydomying, Imogen Koh, Julia Silverii, Aaron Lam, Raymond Zeng, Krisha Solanki, Angus Franks, Liam Edwards, Ridvan Atilhan, Rohan Nandurkar, Oliver Wells, Kristina Vanguardia, Dennis King, Elton Edwards, Liam Edwards, Quang Tran, Michelle Chau, Seojung Min, Abdul Rauf, Yangzirui Fu, Hodo Haximolla, Mengge Shang, Sharrada Segaran, Shelley Wang, Gananadha Sivakumar, Jaspreet Kaur Sandhu, Neel Mishra, Samantha Hauptman, Alyssa Chua, Danielle Chene, Guy Maddern, Henry Shaw, Qiwen Wang, Siyuan Pang, Christine Lu, James Fung, Kathryn Cyr, Karen Lu, Ming Zhou How, Nelson Hu, Paul Anderson, Philip Jakanovski, Arkan Youssef, Howard Tang, Rory Keenan, Alex Chan, Mitch Canny, Farah Tahir, James Egerton, Justin Yeung, Justin Chan, Lea Tiffany, Michael Bei, Mariolyn Raj, Peter Williams, Sakshar Nagpal, Tim Outhred, Russel Krawitz, Colin Chan-Min Choi, Khadijah Younus, Mary Giurgius, Rosemary Kirk, Amanda Gonzalez Pegorer, Pattarapan Tang-Ieam, Jack Ward, Asanka Wijetunga, Caitlin Zhang, Chris Nahm, Christine Wang, Damian Golja, Gregory Jenkins, Helena Qian, Jason Luong, Kim Nguyen, Sean Suttor, Sherman Lai, Vanessa Ma, Yan Chen, Hoi Hang Yu, Amos Lee, Antonio Barbaro, Cameron Mcguinness, Guy Maddern, Stevie Young, Ye Fang Lim, Georgina Trotta, Phoebe Chao, George Ding, Carol Fang, Andi Lu, Prabhath Wagaarachchi, Charlotte Cornwell, Amy Gojnich, Peter Stewart, Isabella Dong, Kenneth Wong, Luca Burruso, Lucinda Hogan, Nathan Mcorist, Ramnik Singh, Ragavi Jeyamohan, Zhen Hou, William Lai, Emily Taylor, Diana Sofia Garces Palacios, Maria Alejandra Nanez Pantoja, Daniel Mauricio Bolanos Nanez, Gilmer Omar Perez Hernandez, Lia Jasmin Jimenez Ramirez, Mohamed Mohamed, Ahmed Kamal El-Taher, Ahmed Elewa, Mahmoud Ayman Soliman, Menna Diab, Radwa Ali, Ahmed Ahmed, Adham Galal, Ahmed Elkhodary, Ali Alaa, Arwa Faisal, Asmaa Badawy, Donia Eldomiaty, Mohamed Al Sayed, Esraa Rasslan, Mohamed Ramadan, Gamal Elsayed Fares, Hashem Altabbaa, Humam Emad, Muneera Alboridy, Mahmoud Mongy, Osama Albarhomy, Osama Selim, Rawan Rafaei, Raneem Atta, Ahmad Altaweel, Yara Sherif, Youssef Elghoul, Yousef Tarek, Ahmed Abdelfatah Sabry, Ahmad Moustafa, Osama AbouHiekal, Osama Al Shaqran, Zeyad Haggag, Ahmed M Abbas, Abdallah Rashad Temerik, Dina Atef, Ahmed Mahmoud, Mahmoud M Saad, Mohammed Ragab, Ahmed Omar Mahmoud, Aya Hussien, Mostafa Abdelbaky, Ismail Muhammad, Afnan Morad, Ahmed Ali, Ahmed Hussien, Ahmed Shipa, Ahmed Aboulfotouh, Ahmed M Abdelaal, Ahmed Mohamed Hashem, Ahmed A Youssef, Ahmed Morsi, Alshymaa Ebrahim, Ahmed M Sayed, Ali Momen Kamel, Abdallah Elmaghrabey, Asmaa Moatasem Elgharib, Amira Abdelrahman, Aml Ali, Samah Abdelnaeam, Asmaa Emam, Amira Gad el-mola, Aya Shaban, Asmaa S Shaltout, Bashayer Nabil, Fady Barsoum, Esraa Mostafa, Doaa Abdelbaset, Doaa Salah, Dina Othman, Safaa Othman, Nour Salah Khairallah, Shimaa Abbas Hassan, Salma Morsi, Armia Azer, Enas Abdelbaset Abdelsamed, Rehab Ahmed, Islam Ibrahim, Esraa AbdElbaset, Esraa Hamoda, Fatma Monib, Fatma Harb, Hager Maher, Hatem Ahmed, Haitham Mohammed, Kerolous Hana, Kerillos Ayoub, Kerollos Henes, Kerollos Shamshoon, Keroles Soliman, Mahmoud Hassanein, Mohamed M Abdelhamid, Magdy Mahdy, Mahmoud Khalil, Manal Ali, Mansour Khalifa, Marwa Amary, Merna Ezz Suliman, Mohamed M Abdallah, Mohammed Saif Al Nasr, Michael Elia, Michael Adly, Mo'men Roshdy, Mohamed F Ramadan, Mohammed A Shahat, Mohammad K Abdelnasser, Moahmed Zaed, Mohammed Al-Quossi, Mohamed A Zarzour, Mohamed M Hares, Mohammed Abdelnasser Abdelfatah, Mahmoud Abughanima, Mahmoud Abdeljaber, Mona Saber, Mostafa K Amin, Mostafa Abbas, Ola Haroon, Omaima Khalil, Omnia Talaat, Rahma Elnagar, Randa Soliman, Reham Aboelela, Salem Salah, Samia Abdelgawad, Tasneem Mohammed, Tarek A Hussien, George Sobhy, Yasmeen Sayed, Yousra Othman Reham Silem, Ali Dawood, Tarek Hemaida, Reem Ahmed, Aya Kamaleldin, Ahmed Zakaria, Mohamed Salah, Ebrahim Salem, Osama Fathy Ali Ali Rashed, Mohamed Halawa, Hossam Elfeki, Abdelrahman Mosaad, Abdelrahman Shaaban, Hebatalla Abdelsalam, Ahmed Sakr, Aly Sanad, Amr Elsawy, Bassant Maged Maged, Dana Hegazy, Mohamed Abdelmaksoud, Mahmoud Laymon, Mohamed Taman, Esraa R Moawad, Hadeer Elsaeed AboElfarh, Karim Elkenawi, Manar Osama, Mirna Sadek, Mohamed Abdelaziz Elghazy, Mohamed Attia, Mohamed Nader, Mostafa Shalaby, Omar Attiya, Osama Samir Gaarour, Ahmed Zaghloul, Pola Mikhail, Karim Badr, Hatem Soltan, Mohamed Donia, Mohammed Gaafar, Khaled Abdelwahab, Abdelaziz Sallam, Ahmed Eid, Mohamed Yousri, Omar Hamdy, Aiman Al-Touny, Abdelrhman Alshawadfy, Ahmed Hamdy, Ahmed Ellilly, Ahmed Mahdy, Ahmed El-Sakka, Hamdy Hendawy, Asmaa Salah, Bassma Raslan, Eman Teema, Eslam Albayadi, Esraa Nasser, Hanaa Mohamed, Mohamed Mahmoud, Mostafa Elsaied, Omima Taha, Shaimaa Dahshan, Shimaa Al-Touny, Ahmed Karrar, Ahmed Khairy, Abdelrahman Farag, Asmaa Deafallah, Alaa Mohamed Ads, Rabiaa Alomar, Issa AbuShawareb, Abdallah Saeed, Abdelhafeez Mashaal, Adel Mohamed Ads, Sohila Ghanem, Ahmed Elghamry, Eman Ayman Nada, Youssef Ali Noureldin, Mohamed Fayez Fouda, Nourhan Shaheen, Shereen Allam, Ibrahim Mazrou, Ali Fahmy Shehab, Wesam Kussaili, Dimitrios Korkolis, Evangelos Fradelos, Aikaterini Sarafi, Nikolaos Machairas, Konstantinos S Giannakopoulos, Fotios Stavratis, Georgios Korovesis, Gerasimos Tsourouflis, Myrto D Keramida, Nikolaos Kydonakis, Stylianos Kykalos, Athanasios Syllaios, Panagiotis Dorovinis, Dimitrios Schizas, Orestis Ioannidis, Anastasia Malliora, Elissavet Anestiadou, Konstantinos Zapsalis, Fotios Kontidis, Lydia Loutzidou, Nikolaos Ouzounidis, Stefanos Bitsianis, Savvas Symeonidis, Smaragda Skalidou, Orestis Ioannidis, Olga Maria Valaroutsou, Themistoklis Dagklis, Alexandra Arvanitaki, Apostolos Mamopoulos, Apostolos Athanasiadis, Stergios Kopatsaris, Ioannis Kalogiannidis, Ioannis Tsakiridis, Georgios Kapetanios, Evangelos Papanikolaou, Nikolaos Tsakiridis, Fotios Zachomitros, Andreas Larentzakis, Argyrios Gyftopoulos, Konstantinos Albanopoulos, Apostolos Champipis, Christos Yiannakopoulos, Gavriella Zoi Vrakopoulou, Konstantinos Saliaris, Konstantinos Lathouras, Spyridon Skoufias, Georgia Doulami, Metaxia Bareka, Eleni Arnaoutoglou, Fragkiskos Angelis, Fragkiskos Angeslis, Michael Hantes, Maria Ntalouka, Maytham A Al-Juaifari, Mohammed Alwash, Rasool Maala, Yasir Adnan Zwain, Sara Ahmed Saleh, Mohammed Khorsheed, Antonio Pesce, Carlo V Feo, Massimiliano Bernabei, Francesca Petrarulo, Nicolò Fabbri, Raffaele Labriola, Silvia Jasmine Barbara, Simone Bosi, Angela Romano, Anna Canavese, Caterina Catalioto, Claudio Isopi, Cristina Larotonda, Gerti Dajti, Matteo Rottoli, Iris Shari Russo, Stefano Cardelli, Francesco Castagnini, Francesco Traina, Giulia Guizzardi, Giulia Giuzzardi, Mara Gorgone, Marco Maestri, Pasquale Cianci, Ivana Conversano, Enrico Restini, Domenico Gattulli, Giorgia Grillea, Marco Varesano, Giacomo Calini, Adelaide Andriani, Davide Gattesco, Giovanni Terrosu, Mattia Zambon, Pietro Matucci Cerinic, Luisa Moretti, Davide Muschitiello, Samantha Polo, Vittorio Bresadola, Salah Abu Wardeh, Mahmoud Al-Baw, Saif Alhaleeq, Subhi Al-Issawi, Esmat Alsaify, Farah Banihani, Noor Massadeh, Nada Massadeh, Dima Al-issawi, Basel Elyan, Qotadah Al-Shami, Yazan Alomari, Almu'atasim Khamees, Sief-Addeen Al-Tahayneh, Ahmad Alsheik, Khaled Sawaftah, Osama Sarhan, Abed Alazeez Alkhatib, Bader Alzghoul, Ahmad Saleh, Jamal Yaghmour, Mahmoud Shahin, Mohammed Maali, Dawood Alatefi, Heba Al-Smirat, Abdulhakim Hezam, Nassar Alathameen, Abdulrahim Al Kaddah, Amr Al Hammoud, Salem Ayasrah, Hamza Abuuqteish, Tesneem Al-Mwajeh, Reena Makableh, Saad Bataineh, Amin Shabaneh, Wesam Alnatsheh, Marwan Aldeges, Huda Hamad, Sireen Shehahda, Dima Khassawneh, Osama Alzyoud, Risan Alrosan, Hasan Awad, Tariq Khaldoon, Rabab Shannaq, Mohammad Al hamoud, Bader Abo fadalah, Mo'ath Al-Hazaimeh, Wail Khraise, Lara Alnajjar, Majjd Alnajjar, Sohaib Al-Omary, Adnan Ababneh, Alaa Albashaireh, Mohammad Khadrawi, Mohammad Aljamal, Tayseer Athamneh, Ro-a Muqbel, Maryam Al-jammal, Ahmad Masarrat, Alia Al-zawaydeh, Ibrahim Taha, Taima’ Qattawi, Rayyan Smadi, Ayah Alhaleem, Mosab Alboon, Omar Hazaymeh, Leen Karasneh, Safa’ Al-Haek, Marin Almahroush, Tamam Alfrijat, Aya Elporgay, Hadeel Shanag, Hamza Agilla, Hind Alameen, Marya Bensalem, Mawadda Altair, Malak Ghemmied, Rehab Alarabi, Sara Alhudhairy, Rima Gweder, Amal Alzarroug, Ebtihal Alabed, Fadwa Elreaid, Omar A Elkharaz, Fatma Fathi Elreaid, Safa Sasi Albatni, Haitham Elmehdawi, Milad Gahwagi, Ayman Mohamed, Tariq Alfrjani, Khaled Khafifi, Ayat Rasheed, Ayoub Akwaisah, Hassan Bushaala, Mustafa Elfadli, Mohamed Moftah, Salima Algabbasi, Salma Esaiti, Sara Elfallah, Abtisam Alharam, Fatima Alariby, Mohamed Isweesi, Tarik Ahmed Eldarat, Ayman Arhuma Dabas, Akram Alkaseek, Ahmed Mohammed Abodina, Aya Alqaarh, Hibah Bileid Bakeer, Hoda Salem Alhaddad, Husein Aboudlal, Sawsan Alsaih, Noora Abubaker, Najwa Abdelrahim, Ali Alzarga, Basma Omar, Farah Faris, Qamrah Alhadad, Asma Abufanas, Hussameddin Badi, Israa Benismai, Hawa Obeid, Abdulwahab Abdalei, Ahmed Abdulrahman, Aisha Swalem, Ebtisam Alzarouq, Amna Safar, Esra Shagroun, Boshra Hashem, Fatheia Elrishi, Fatima Abdulali, Habeeba Ahmed, Ibrahim Eltaib, Joma Elzoubia, Aisha Albarki, Hoda El Mugassabi, Fatima Abushaala, Amany Abuzaho, Nida Juha, Raneem Egzait, Sundes Shetwan, Alzahra Lemhaishi, Faisel Matoug, Eman Abdulwahed, Aamal Askar, Abir Ben Ashur, Adel Bezweek, Bushra Altughar, David Emhimmed, Donia Elferis, Laila Elgherwi, Enas Soula, Doaa Gidiem, Maren Grada, Khawla Derwish, Maram Alameen, Nassib Algatanesh, Ahlam Elkheshebi, Reem Ghmagh, Sharf Barka, Sultan Ahmeed, Sarah Aljamal, Zahra Alragig, Mohamed Addalla, Ahmed Atia, Atab Kharim, Fathia Mahmoud, Muhannud Binnawara, Entisar Alshareea, Mohamed Alsori, Aisha Alshawesh, Ghaliya Mohamed H Alrifae, Amira Ashour, Anwaar Abozid, Asil Omar Saleh Alflite, Anwar Mohamed, Jaber Arebi, Fatma Alagelli, Hana Yousef Gineeb, Rawia Ghmagh, Rihab Mohammed Bin Omar, Retaj Alaqoubi, Sara Mohammed, Serien Hossain Bensalem, Tahani Elgadi, Wesam Sami, Yara Bariun, Abdulhadi Mohammed Alhadi Alhashimi, Dheba Almukhtar Abdulla, Heba Rhuma, Husam Enaami, Asraa Ali Alboueishi, Hayat Ben Hasan Mohamed A A Alkchr, Bashir Abobaker Albakosh, Najat Ben Hasan, Najah Alsari, Mohammed Aldreawi, Khaled Abushanab, Rawad Yahya, Narimantas Samalavicius, Vitalijus Eismontas, Jonas Jurgaitis, Oleg Aliosin, Vitalija Nutautiene, Andee Dzulkarnaen Zakaria, Anil Kumar Sree Kumar Pillai, Dinesh Kumar Vadioaloo, Mohamed Ashraf Mohamed Daud, Jien Yen Soh, Mohd Zaim Zakaria, Siti Mayuha Rusli, Nur Ayuni Khirul Ashar, Zatul Akmar Ahmad, Afiq Aizat Ramlee, Sharifah Nor Amirah Syed Abdul Latiff Alsagoff, Ahmad Anuar Sofian, Muhammad Badrul Hisyam Mohamad Jamil, Bahiyah Abdullah, Mohamad Faiz Noorman, Muhammad Fihmi Zainal Abidin, Mohamed Izzad Isahak, Siti Nasyirah Nisya Adnan, Zaidatul Husna Mohamad Noor, Luis Adrian Alvarez-Lozada, Alejandro Quiroga Garza, Andrea Aguilar Leal, Bernardo Alfonso Fernández Reyes, Ethel Valeria Orta Guerra, Francisco Javier Arrambide Garza, Héctor Erasmo Alcocer Mey, Jorge Arath Rosales Isais, Juventino Tadeo Guerrero Zertuche, Patricia Ludivina González García, Luis Antonio Heredia Sánchez, Marcela Patricia Flores Mercado, Oscar Alonso Verduzco Sierra, Pedro Emiliano Ramos Morales, Stephie Oyervides Fuentes, Víctor Manuel Peña Martínez, Yesika Alejandra Guerra-Juárez, Ana Karina Flores-González, Surya Singh, Arwa Hadi, Christian Woodbridge, David Thornton-Hume, Jack Forsythe, Isini Dharmaratne, Vivian Pai, John Windsor, Kamran Zargar, Lucy Waldin, Lily Winthrop, Matias Alvarez, Meileen Huang, Matt Kumove, Marta Simonetti, Namisha Chand, Oliver Goldsmith, Oscar Guo, Paul Monk, Karen Zhou, Sai Harshitha Penneru, Shaamnil Prasad, Seifei Ren, Terrence Hill, Vyoma Mistry, Selena Sun, Ashley Pereira, Scott Mclaughlin, Andrew Stokes, Avinash Sathiyaseelan, Jeremy Rossaak, Janice Lim, Kenya Brooke, Liam Quinlan, Mark Pottier, Nayanika Podder, Puja Jinu, Shanay Ramphal, Wikus Vermeulen, Flavio Ordones, Fraser Jeffery, Ibrahim S Al Busaidi, Janelle Divinagracia, William Ju, Yizhuo Liu, Tamara Glyn, Nasya Thompson, Vivien Graziadei, Joshua Canton, Joseph Furey, Horim Choi, Grace Coomber, Tanya Divekar, Tessa English, Erin Gernhoefer, Tom Healy, Justin Chou, Dikshya Parajuli, Catherine Reed, Rod Studd, Anthony Lin, Cameron Wells, Cindy Xu, Arwa Hadi, Andrew Maccormick, Heejun Park, Athulya Rathnayake, Brittany Williams, Ashley Chan, Corinne Smith, Francesca Casciola, Jainey Bhikha, Jonathan Luo, Kevin Yi, Megan Singhal, Ria George, Rosie Luo, Taylor Frost, Fatima Hakak, Akhita George, Angela Carlos, Annie Ho, Connor Mcrae, Jonathan Lescheid, Jenny Soek, Andrew Pham, Sophie St Clair, Su-Ann Yee, Jennifer Lim, Chun-Yen Wu, Taehoon Kim, Anne Qi Chua, Christopher Harmston, Hamish Boyes, Holly Cook, Jamie Struthers, Jess Radovanovich, Nicholas Quek, Chekodi Fearnley-Fitzgerald, Deborah Wright, Kushan Ghandi, Natalie Matheson, Matthew James McGuinness, Brian Chen, Rebecca Indiana Douglas, Konrad Richter, Nisha Bianca Soliman, Scott Matthew Bolam, Vineeth Vimalan, William Currie, Mitchell Cuthbert, Poppy Ross, Amy Nicholson, Briar Garton, Emilie Agnew, Niamh Conlon, Nicholas Waaka, Ritwik Kejriwal, Sean Nguyen, Edmund Leung, Milidu Ratnayake, Quintin Smith, Nejo Joseph, Bosco Yue, Calvin Fraser, Charles Lam, Ethan Figgitt, Gordon Liu, Kevin Tan, Ha Seong You, Helen Zheng, Jenny Luo, James Sharp, Kabir Khanna, Levi Simiona, Michel Luo, Milidu Ratnayake, Patrick Wong, Rebecca Luu, Rohit Paul, Shiva Nair, Shadie Asadyari-Lupo, Wing Hung, Geoffrey Ying, Jess Ho, Alan Wu, Eamon Walsh, Jouyee Lee, Jessie Liu, Sunny Yao, Omar Nosseir, Jennifer Dang, Simon Young, Sof'ya Zyul'korneeva, Theresa Boyd, Jess Ho, Alan Wu, Sunny Yao, Abdullahi Musa Kirfi, Adamu Bala Ningi, Mohammad Albuhari Garba, Makama Baje Salihu, Ohia Ernest Ukwuoma, Abdullahi Ibrahim, Isa Mienda Sajo, Muhammad Baffah Aminu, Liman Haruna Usman, Oloko Nasirudeen Lanre, Ibrahim Shaphat Shuaibu, Stephen Yusuf, Tiamiyu Ismail, Gabi Ibrahim Umar, Ademola Adeyeye, Ehis Afeikhena, Favour Chinenye Nnaji, Joy Onyekachi Agu, Temiloluwa Peace Maxwell, Oluwatosin Olakunle Motajo, Oghenekaro Ifoto, Seubong-Abasi Imoh Okon, Jerry Godfrey Makama, Amina Abosede Mohammed-Durosinlorun, Bashiru Aminu, Polite Iwedike Onwuhafua, Caleb Mohammed, Lubabatu Abdulrasheed, Joel Amwe Adze, Khadijah Richifa Suleiman, Lydia Regina Airede, Mathew Chum Taingson, Stephen Bodam Bature, Stephen Akau Kache, Uchechukwu Ohijie Ogbonna, Mohammed Bello Fufore, Abdulkarim Iya, Adeshina A Ajulo, Ahmad Mahmud, Bilal Shuaibu Yahya, Farida Onimisi-Yusuf, Hope Isaac, Timothy Jawa, Fashe Joseph, Bemi Kala, Maisaratu A Bakari, David Wujika Ngwan, Abubakar umar, Abraham L Filikus, Daniel Wycliff, Abiodun Okunlola, Olukayode Abiola, Adebayo Adeniyi, Olabisi Adeyemo, Babatunde Awoyinka, Olakunle Babalola, Adewumi Bakare, Taiwo Buari, Cecilia Okunlola, Gbadebo Adeleye, Adedayo Salawu, Henry Abiyere, Adetolu Ogidi, Tesleem Orewole, Habiba Ibrahim Abdullahi, Godwin Akaba, Arome Achem, Asi-oqua Bassey, Emeka Ayogu, Bilal Sulaiman, Dennis Anthony Isah, Chukwunonso Nnamdi Akpamgbo, Felicia Asudo, Nathaniel Adewole, Omachoko Oguche, Peter Ejembi, Samuel Ali Sani, Paul Chimezie Andrew, AliyuYabagi Isah, Bolarinwa Eniola, Zumnan Songden, Teddy Agida, Terkaa Atim, Taofiq Olayinka Mohammed, Hadijat Olaide Raji, Femi Ibiyemi, Hafeez Salawu, Olushola Fasiku, Remi Sanyaolu Solagbade, Mariam Motunrayo Shiru, Gbadebo Hakeem Ibraheem, Justina Oruade, Grace Ezeoke, Tabish Chawla, Aliya Begum Aziz, Anoosha Marium, Ayesha Akbar Waheed, Faiqa Binte Aamir, Faiza Qureshi, M Hammad Ather, Iqra Fatima Munawar Ali, Izza Tahir, Maha Ghulam Akbar, Ronika Devi Ukrani, Sajjan Raja, Sehar Salim Virani, Shahryar Noordin, Saif Ur Rehman, Shalni Golani, Syed Roohan Aamir, Syed Musa Mufarrih, Usama Waqar, Maliha Taufiq, Ahmed Siddique Ammar, Adya Ejaz, Albash Sarwar, Ahmed Usman Khalid, Shehrbano Khattak, Aliza Imran, Omer Bin Khalid, Urauba Kaleem, Urwah Muneer, Yumna Kashaf, Fatima Zafar, Adil Zaheer, Muhammad Ali, Amna Shafaat, Arisha Qazi, Asjad Imran,Mahnoor Tariq, Muhammad Nadeem Aslam, Shehroz Ali, Tabish Atiq, Tayyiba Wasim, Daniyal Babar, Ahmad Zain, Muhammad Ibtisam, Uzair Ahmed, Syed Talha Bin Aqeel, Muhammad Muhib, Muhammad Anas Abbal, Nasar Ahmad Khan, Imran Javed, Layth Alkaraja, Dana Amro, Ghaida Manasrah, Ibraheem Hammouri, Ihab Abu Hilail, Jihad Zalloum, Laith Alamlih, Mahmoud Nasereddin, Munia Rajabi, Sa'ed Shalalfeh, Zeinab Natsheh, Khamis Elessi, Mustafa Abu Jayyab, Mohammed Astal, Mosheer Al-Dahdouh, Alaa Eddin Salameh, Alaa Ayyad, Nimatee Dawod, Hamza Alsaid, Iyas Matar, Majd Hassan, Mohammed Bakeer, Mohammad Malasah, Shehab Abuhashem, Mohammed Salem, Sorinel Lunca, Mihail Gabriel Dimofte, Stefan Morarasu, Ana Maria Musina, Cristian Ene Roata, Natalia Velenciuc, Aleksandr Butyrskii, Maxim Bozhko, Amet Ametov, Sharfuddin Chowdhury, Doaa Bagazi, Julio Domenech, Alejandro Rosello-Añon, Ana Monis, Caterina Chiappe, Beatriz Cuneo, Pablo Clemente-Navarro, Jorge Febre, Jorge Sanz-Romera, Marcos Lopez-Vega, Ignacio Miranda, Rocio Valverde-Vazquez, Sara Garcia, Maria Jose Sanguesa, Zutoia Balciscueta, Enrique Ruiz, Eduardo Marco, Elena Talavera, Joan Farre, Loreto Bacariza, Mireia Duart, Violeta Ureña, Xenia Carre, Hytham K S Hamid, Montasir A Abd-Albain, Sami Galal-Eldin, Monira Sarih, Eithar Adam, Samir Ismail, Malaz Azhari, Tawfieg Hassan, Mohamed Salaheldein, Zainab Abdalla, Wahiba Ahmed, Monzer Alhassan, Abdulatif Mohamed, Hozifa Mohamed Abdalla Suliman, Mohammed Omer Mohammed Eltayeb, Rogia Ahmed Abdalla Ahmed, Enas Mohammedtom Abdulhameed Babekir, Munya Ali Talab Khairy, Maha Mukhtar Ahmed Mukhtar, Rzan Ali Hamedelneel Ali, Yasir Babkir Ali Al-Shambaty, Fatima Imad Yousif, Hawa Mohammed Hassan Mohammed, Lana Osher, Lana Osher, Menhag Abdelbast, Mohamed Yassin, Noon Moawia, Rowa Abdalsadeg, Abrar Husein, Baraa Elhassan, Alnazeer Y Abdelbagi, Mohammed A Adam, Eithar M Ali, Ibrahim A b Mohammed, Maab Mohamed, Mohamed Abdulaziz, Mazin Akasha, Muaz Hassan, Nadir Hilal, Noon Abdalla Abdelrahman Mohamed, Noora Abubaker, Omeralfarouk Mohammed, Shakir Mohamed, Walaa Osman, Fatima Mustafa, Alaa A Salih, Doua Ali, Doha Mohammed Ahmed Almakki, Hanan Elnour Mohamed, Abdelhadi Elmubark, Mohamed Hassan, Ammar Alnour, Amna Elaagib, Ayman Abdelrahman, Mubarak Abdelkhalig, Khalid Nour Eldaim, Afra Babiker, Entisar Ahmed, Maab Ali, Eman Hussain, Mansour Wedatalla, Alaaaldeen Ahmed, Alla Aldeen Hamza, Mohab Mohammed, Omer Osman, Reham Ibrahim, Rihab Ahmed, Ruaa Ahmed, Ruaa Yasir, Safaa Awadallah, Sara Mohmmed, Suhaib Hassan, Walid Shaban, Aisha Hussein, Reem Rafea, Ahmed Abdalla, Abdalla Ahmed, Khalid Mohamed, Mansour Mohammed, Mohamed Altahir, Mohammed Adam, Omer Mohamed, Walaa Abdullah, Hammad Fadlalmola, Ahmed Yassir Abdalla, Ahmed Ali Omer, Ahmed Alfatih Mustafa, Rawan Elnoman Hamadelniel Elhadi, Essam Eldien Abuobaida Banaga, Fatima Osman, Mohamed Galal Ali Abdalla, Hala Abdelhalim Mohamed Taha, Noon Ezzeldien Abdalmahmoud, Rofuida Hussien Nafie, Sami Jamal, Sharwany Ahmed, Rawan Alsheikh Ali, ‏Abdallah ‏Aladna, ‏Abdullah ‏Aljoumaa, ‏Hamdi ‏Nawfal, Salma Jamali, ‏Fatima Khouja, ‏Ammar ‏Niazi, Toka ‏ ‏ Al Rawashdeh, Nahla Kechiche, Mouna Gara, Mouna Nasr, Marwen Baccar, Oumayma Benamor, Sawssen Chakroun, Ahmet Necati Sanli, Ahmet Yildiz, Mehmet Ali Demirkiran, Yildiz Buyukdereli Atadag, Yusuf Iskender Tandogan, Esin Ozkan, Yıldırım Ozer, Esin Ozkan, Muhammed Miran Oncel, Senad Kalkan, Tolga Gover, Berke Manoglu, Ilayda Oksak, Ipek Kurt, Kerem Rifaioglu, Selman Sokmen, Tayfun Bisgin, Yasemin Yildirim, Abdil Yetkin Keskin, Tugce Dogan, Berfin İlgaz Sahin, Cemil Aydin, Duygu Ece Benek, Hale Nur Tiras, Mert Arslangilay, Mert Aslangilay, Muhammet Yaytokgil, Mehmet Ali Capar, Yasemin Yazgan, Sebnem Bektas, Ahmet Can Alagoz, Alara Ece Dagsali, Aylin Izgis, Kadir Uzel, Mustafa Soytas, Niyazi Cakir, Abdullah Emre Askin, Ibrahim Azboy, Kubilay Sabuncu, Merve Aslan, Melek Sahin, Mustafa Oncel, Nuri Okkabaz, Ramazan Kemal Sivrikaya, Alparslan Saylar, Alparslan Saylar, Meltem Yasar, Ergin Erginoz, Haktan Ovul Bozkir, Kagan Zengin, Mehmet Faik Ozcelik, Server Sezgin Uludag, Zeynep Ozdemir, Osman Sibic, Hatice Telci, Mehmet Abdussamet Bozkurt, Yasin Kara, Mustafa Deniz Tepe, Adnan Gündoğdu, Bilge Akın, Dilan Pehlivan, Ali Guner, Duygu Baysallar, Berkay Yıldız, Hale Cepe, Murat Emre Reis, Ayse Nilufer Yuzgec, Nurtac Kıralı, Taha Anıl Kodalak, Mehmet Ulusahin, Kamar Selim, Ahmet Kale, Mehmet Emre Gecici, Melis Ozbilen, Zeynep Düzyol, Aylin Gemici, Elzem Korkmaz, Eminenur Şen, Muhammed Enes Taşcı, Elifsu Camkıran, Güşta Elieyioğlu, İkbal Kayabaş, Tevfik Kıvılcım Uprak, Canan Aral, Ayten Saraçoğlu, Mustafa Ümit Uğurlu, Zeynep Hazal Baltacı, Ege Nur Akkaya, Cem Fergar, Elif Zeynep Tabak, Guldane Zehra Kocyigit, Ilgaz Kayilioglu, Süleyman Polat, Eli˙f Çolak, Mehmet Emin Kara, Mert Candan, Mustafa Safa Uyanık, Ahmet Can Sarı, Attila Ulkucu, Alperen Taha Certel, Arzu Dindar, Beyza Durdu, Cigdem Bayram, Eslem Kaya, Hakan Akdere, Ibrahim Ethem Cakcak, Ikranur Yavuz, Mert Omur, Mirac Ajredini, Erhan Onur Aydoğdu, Eylül Şenödeyici, Ulku Ceren Koksoy, Baturay Kansu Kazbek, Deniz Serim Korkmaz, Dogancan Yavuz, Hakan Yilmaz, Zeynep Sahan Cetınkaya, Elif Durmus, Filiz Tuzuner, Furkan Hokelekli, Mucahid Mutlu, Seyma Orcan Akbuz, Ziya Can Kus, Ziya Can Kus, Michael Farrell, Alayna Craig-Lucas, Matthew Painter, Ashley Titan, Aditya Narayan, Bunmi Fariyike, Lisa Knowlton, Tiffany Yue, Emily Benham, Abdelrahman Nimeri, Hope Werenski, Nicole Kaiser, Caroline Reinke

## Abstract

**Background:**

Balancing opioid stewardship and the need for adequate analgesia following discharge after surgery is challenging. This study aimed to compare the outcomes for patients discharged with opioid *versus* opioid-free analgesia after common surgical procedures.

**Methods:**

This international, multicentre, prospective cohort study collected data from patients undergoing common acute and elective general surgical, urological, gynaecological, and orthopaedic procedures. The primary outcomes were patient-reported time in severe pain measured on a numerical analogue scale from 0 to 100% and patient-reported satisfaction with pain relief during the first week following discharge. Data were collected by in-hospital chart review and patient telephone interview 1 week after discharge.

**Results:**

The study recruited 4273 patients from 144 centres in 25 countries; 1311 patients (30.7%) were prescribed opioid analgesia at discharge. Patients reported being in severe pain for 10 (i.q.r. 1–30)% of the first week after discharge and rated satisfaction with analgesia as 90 (i.q.r. 80–100) of 100. After adjustment for confounders, opioid analgesia on discharge was independently associated with increased pain severity (risk ratio 1.52, 95% c.i. 1.31 to 1.76; *P* < 0.001) and re-presentation to healthcare providers owing to side-effects of medication (OR 2.38, 95% c.i. 1.36 to 4.17; *P* = 0.004), but not with satisfaction with analgesia (β coefficient 0.92, 95% c.i. −1.52 to 3.36; *P* = 0.468) compared with opioid-free analgesia. Although opioid prescribing varied greatly between high-income and low- and middle-income countries, patient-reported outcomes did not.

**Conclusion:**

Opioid analgesia prescription on surgical discharge is associated with a higher risk of re-presentation owing to side-effects of medication and increased patient-reported pain, but not with changes in patient-reported satisfaction. Opioid-free discharge analgesia should be adopted routinely.

## Teaser

This international, multicentre, prospective cohort study of 4273 patients from 25 countries undergoing common acute and elective general surgical, urological, gynaecological, and orthopaedic procedures found that opioid prescription on discharge was not associated with decreased pain or increased satisfaction with analgesia after discharge. Although opioid prescribing varied dramatically between high-income and low- and middle-income countries, patient-reported outcomes did not.

## Introduction

Postoperative pain is common, complex, and often severe, affecting up to 80% of patients^1^. It is frequently managed using opioid analgesia, which, although potent, incurs the risk of significant adverse events. There are ongoing concerns regarding the inappropriate, excessive, and unsafe prescription of opioid analgesia after surgery^2,3^. Such practices are associated with worse patient outcomes, greater healthcare demands, and the ‘opioid epidemic’^4,5^.

Surgeons are high prescribers of opioids, accounting for approximately 10% of all prescriptions^6^. The overprescription of opioids in the postoperative phase likely stems from healthcare providers’ desire to reduce patient discomfort, and concerns regarding poor patient satisfaction^7,8^. Unused postoperative opioids represent an important contributor to opioid diversion in the community^9^. Despite the perception that opioids aid pain management in this setting, multiple studies have indicated that opioid-sparing protocols after surgery are not associated patient satisfaction, provided that pain is controlled adequately using non-opioid analgesia, and patient expectations are managed appropriately^7,10^.

A recent meta-analysis by Fiore *et al.*^11^ reported that opioid prescription at discharge after elective procedures did not improve pain control, but was associated with increased harm. This review was limited to minor and moderate procedures such as dental procedures or cholecystectomies, but demonstrated that the default stance to prescribe opioids at surgical discharge may be a practice steeped in culture rather than evidence. Given the variation in prescribing practices between countries, centres, and individual clinicians^12^, international, multispecialty data are required to understand the relationship between opioid prescription and patient-reported pain and satisfaction outcomes, particularly after emergency and major procedures.

This study aimed to describe patient-reported outcomes of patient pain, satisfaction, and quality of life after common surgical procedures, and to investigate the effect of opioid prescription on patient-reported postdischarge outcomes and the risk of re-presentation owing to inadequate analgesia or adverse effects.

## Methods

### Study design

The OPERAS (Opioid PrEscRiptions and Usage After Surgery) study was an international multicentre collaborative study developed by the Trials and Audit in Surgery by Medical Students in Australia and New Zealand (TASMAN) Collaborative, an Australasian student- and trainee-led collaborative network. The study was registered with the Australian New Zealand Clinical Trials Registry (ACTRN12621001451897p) and the protocol (*Appendix S1*) has been published elsewhere^13^. The collaborative research model has been used by other studies internationally and described previously^14,15^. Study requirements and approvals were achieved according to country-specific regulations before recruitment of participants began. This analysis was conducted in line with the STROBE reporting guidelines for observational studies^16^. Patients or the public were not involved in the design, conduct, reporting or dissemination plans of this research.

### Ethical approval

Ethical approval was obtained at each participating site in line with local protocols and verified by the central steering committee. The protocol was approved by the Hunter New England Human Research Ethics Committee (2021/ETH11508) in Australia as the lead site.

### Eligibility criteria

Adult patients aged 18 years or above who underwent an eligible general surgical (cholecystectomy, appendicectomy, inguinal hernia repair, colonic resection, fundoplication or sleeve gastrectomy), orthopaedic (total or reverse shoulder arthroplasty, rotator cuff or labral repair, anterior cruciate ligament repair, or hip or knee arthroplasty), gynaecological (hysterectomy, oophorectomy, or salpingectomy and oophorectomy), or urological (prostatectomy, cystectomy or nephrectomy) operation during the data collection phases were approached for inclusion^13^. Elective and emergency operations were included. Patients who fulfilled any of the following criteria were excluded: currently on medication-assisted treatment of opioid dependence; discharged to another healthcare setting (for example rehabilitation service); multivisceral resection; and returned to the operating room during index admission. Eligible patients were identified through inspection of surgical operating lists. All participants provided written informed consent before inclusion and each centre obtained ethical approval before data collection.

### Data collection

Data were collected prospectively during six separate 2-week intervals between April and September 2022, from inpatient hospital records and by a follow-up telephone call 7 days after discharge. The follow-up interview was conducted using a prespecified protocol and script (*Appendix S2*) to ensure standardization.

Data were collected on patient demographics (age, sex, smoking status, BMI, ASA physical status grade), co-morbidities, diagnosis and procedure-specific details (indication, surgical approach, and urgency), opioid use in the 24 h before discharge from hospital, postoperative complications, and preoperative regular opioid and non-opioid analgesic use. Regular analgesic use was defined as a minimum of once-weekly use in the 3 months immediately before surgery. Minimally invasive surgery was defined as arthroscopic, laparoscopic or robotic surgery. Open surgery was defined as planned open surgery, and laparoscopic/robotic operations that were converted to open procedures. Data were also collected on opioid and non-opioid medication prescriptions at discharge.

To enable comparison between opioids of different potencies, all data on opioid doses were converted to oral morphine equivalents (OMEs). Further details on how OMEs were calculated are provided in *Appendix S3*.

### Outcomes

The primary outcome was the amount of time spent in severe pain in the first 7 days after discharge measured on a numerical analogue scale from 0 to 100%. Secondary outcomes included patient-reported satisfaction with the quality of analgesia received on a scale of 0 to 100, patient-reported quality of life measured by the EQ-5D-5L™ tool (EuroQol Group, Rotterdam, the Netherlands) 7 days after discharge, number of presentations to healthcare professionals owing to inadequate analgesia, and number of presentations to healthcare because of side-effects of analgesia including nausea, vomiting, drowsiness, itching, dizziness or constipation. Outcome measures related to the prescription and consumption of analgesics are reported elsewhere^17^.

The EQ-5D-5L™ tool is used to measure patient quality of life in five domains, including mobility, self-care, usual activities, pain/discomfort, and anxiety/depression. The tool also includes an EQ-VAS score, which rates a patient’s self-reported health from 0 (worst possible) to 100 (best possible)^18^. Re-presentation to healthcare was defined as any visit to primary care, emergency department, surgeon’s office, or readmission to hospital for inadequately treated pain or side-effects of analgesic medication between discharge and 7 days after discharge.

### Statistical analysis

Statistical analyses were completed in R 4.0.3 for statistical computing (R Core Team, Vienna, Austria). Descriptive statistics were used to compare demographic and in-hospital differences between patients discharged on opioid and opioid-free analgesia using χ^2^ tests for categorical variables and Kruskal–Wallis tests for continuous variables.

Propensity score matching was used to minimize the selection bias of participants prescribed opioid and opioid-free analgesia on discharge using the *MatchIt* package^19^. The propensity score was defined as the probability that a patient would receive opioid analgesia adjusted for age, sex, ASA grade, BMI, presence of chronic kidney disease and liver disease, smoking status, preoperative opioid and non-opioid analgesia use, surgical procedure, duration of operation, indication for surgery, postoperative complications, duration of hospital stay, concomitant discharge prescription of paracetamol and non-steroidal anti-inflammatory drugs, and OMEs consumed in the 24 h before discharge. Full matching was used to allow multiple patients from each group to be matched together (if appropriate) and weighted for balancing, avoiding inappropriate discarding of data that can occur with nearest-neighbour matching^20^. Balance between groups was assessed before and after matching using the standardized mean difference, with an absolute value of less than 0.1 as an indication of a well balanced variable.

Multiple imputation by chained equations was used to impute values for patients with missing data using the *mice* package^21^. Visual inspection of variables with missing data stratified by presence of opioid analgesia at discharge was completed to ensure that variables were missing at random. Ten imputed data sets were created, with propensity score matching subsequently performed on each using the *MatchThem* package^22^. The pooled results of models are presented.

Mixed-effects models, using centre and country as random effects, were fitted for primary and secondary outcomes. Logistic regression models were built for binary outcomes, and negative binomial regression or generalized linear regression models for continuous outcomes. Co-variate selection was guided by clinical plausibility and previous literature^23^, and relevant preoperative, intraoperative, and postoperative variables were included as fixed effects. Residual, Q-Q plots, and variance inflation factors were interrogated to assess model assumptions.

#### Subgroup analysis

Procedures were stratified into abdominal procedures (including general, gynaecological, and urological operations) and orthopaedic procedures. Sensitivity analyses were undertaken for these subgroups. Prescribing practices and patient-reported outcomes were compared between different regions of the world, and between high-income countries (HICs) and low- and middle-income countries (LMICs) as defined by the Organisation for Economic Co-operation and Development (OECD)^24^.

## Results

**Fig. 1:**
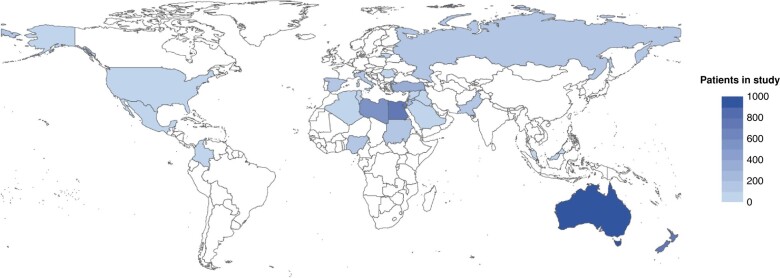
Patients contributed to the OPERAS study by country Full details are available in *Table S1*.

Data from 4273 patients from 114 hospitals in 25 countries were collected over the study interval, (*Fig. 1*). The majority of patients included were from Australia (813), Egypt (594), Aotearoa New Zealand (560), Libya (372), and Turkey (296) (*Table S1*). Patient demographics and co-morbidities stratified by opioid analgesia prescription on discharge are presented in *Table 1* and *Table S2*. The median age of the cohort was 50 years, and 53.1% were women. Of those included, 3056 (71.5%) underwent general surgical procedures, 591 (13.8%) orthopaedic procedures, 393 (9.2%) gynaecological procedures, and 233 (5.5%) urological procedures (*Table 1*).

**Table 1:** Patient characteristics

	No opioids (*n* = 2962)	Opioids (*n* = 1311)	Total (*n* = 4273)	P*
Age (years), median (i.q.r.)	48 (33–64)	52 (37–66)	50 (34–64)	< 0.001†
**Sex**				0.033
Female	1579 (53.3)	692 (52.8)	2271 (53.1)	
Male	1383 (46.7)	616 (47.0)	1999 (46.8)	
Other	0 (0)	3 (0.2)	3 (0.1)	
**ASA fitness grade**				< 0.001
I	1377 (46.5)	386 (29.4)	1763 (41.3)	
II–III	1559 (52.6)	907 (69.2)	2466 (57.7)	
IV—V	24 (0.8)	15 (1.1)	39 (0.9)	
Missing	2 (0.1)	3 (0.2)	5 (0.1)	
**BMI (kg/m^2^)**				< 0.001
Normal (18.5–24.9)	835 (28.2)	327 (24.9)	1162 (27.2)	
Overweight (25.0–29.9)	1078 (36.4)	385 (29.4)	1463 (34.2)	
Obese (30.0-39.9)	603 (20.4)	333 (25.4)	936 (21.9)	
Severely obese (>40.0)	111 (3.7)	94 (7.2)	205 (4.8)	
Underweight (< 18.5)	56 (1.9)	13 (1.0)	69 (1.6)	
Missing	279 (9.4)	159 (12.1)	438 (10.3)	
**Smoking status**				< 0.001
Never smoked	1841 (62.2)	723 (55.1)	2564 (60.0)	
Current smoker	550 (18.6)	182 (13.9)	732 (17.1)	
Ex-smoker	355 (12.0)	307 (23.4)	662 (15.5)	
Missing	216 (7.3)	99 (7.6)	315 (7.4)	
**Procedure**				< 0.001
Appendicectomy	522 (17.6)	241 (18.4)	763 (17.9)	
Cholecystectomy	880 (29.7)	346 (26.4)	1226 (28.7)	
Colorectal resection	271 (9.1)	122 (9.3)	393 (9.2)	
Inguinal hernia repair	440 (14.9)	136 (10.4)	576 (13.5)	
Nissen fundoplication	23 (0.8)	5 (0.4)	28 (0.7)	
Sleeve gastrectomy	49 (1.7)	21 (1.6)	70 (1.6)	
ACL repair	57 (1.9)	21 (1.6)	78 (1.8)	
Knee arthroplasty	110 (3.7)	147 (11.2)	257 (6.0)	
Hip arthroplasty	110 (3.7)	91 (6.9)	201 (4.7)	
Rotator cuff repair	11 (0.4)	11 (0.8)	22 (0.5)	
Shoulder arthroplasty	9 (0.3)	11 (0.8)	20 (0.5)	
Shoulder labral repair	12 (0.4)	1 (0.1)	13 (0.3)	
Cystectomy	28 (0.9)	4 (0.3)	32 (0.7)	
Nephrectomy	62 (2.1)	38 (2.9)	100 (2.3)	
Prostatectomy	66 (2.2)	35 (2.7)	101 (2.4)	
Hysterectomy	228 (7.7)	59 (4.5)	287 (6.7)	
Oophorectomy and salpingectomy	28 (0.9)	13 (1.0)	41 (1.0)	
Oophorectomy only	21 (0.7)	3 (0.2)	24 (0.6)	
Salpingectomy only	35 (1.2)	6 (0.5)	41 (1.0)	
**Surgical approach**				< 0.001
MIS	1487 (50.2)	814 (62.1)	2301 (53.8)	
Open	1474 (49.8)	496 (37.8)	1970 (46.1)	
Missing	1 (0.0)	1 (0.1)	2 (0.0)	
**Indication**				0.630
Benign disease	2601 (87.8)	1144 (87.3)	3745 (87.6)	
Malignancy	360 (12.2)	167 (12.7)	527 (12.3)	
Missing	1 (0.0)	0 (0)	1 (0.0)	
**Urgency**				< 0.001
Elective	2098 (70.8)	818 (62.4)	2916 (68.2)	
Emergency	863 (29.1)	493 (37.6)	1356 (31.7)	
Missing	1 (0.0)	0 (0)	1 (0.0)	
Duration of surgery (min), median (i.q.r.)	80 (55–120)	98 (66–135)	87 (60–120)	< 0.001†
**Opioid use before surgery**				< 0.001
No	2909 (98.2)	1180 (90.0)	4089 (95.7)	
Yes	53 (1.8)	131 (10.0)	184 (4.3)	
**Non-opioid analgesia use before surgery**				< 0.001
No	2396 (80.9)	933 (71.2)	3329 (77.9)	
Yes	566 (19.1)	378 (28.8)	944 (22.1)	
**Clavien–Dindo grade of complication**				< 0.001
None	2450 (82.7)	1025 (78.2)	3475 (81.3)	
I–II	487 (16.4)	265 (20.2)	752 (17.6)	
III–IV	21 (0.7)	20 (1.5)	41 (1.0)	
Missing	4 (0.1)	1 (0.1)	5 (0.1)	
Duration of hospital stay (days), median (i.q.r.)	2 (1–3)	2 (1–3)	2 (1–3)	0.698†
OMEs consumed 24 h before discharge, median (i.q.r.)	0.0 (0.0–30.0)	35.0 (10.0–67.8)	9.0 (0.0–40.0)	< 0.001†

Values are *n* (%) unless otherwise indicated. ACL, anterior cruciate ligament; MIS, minimally invasive surgery; OME, oral morphine equivalent. *χ^2^ test, except †Kruskal–Wallis test.

A total of 1311 patients (30.7%) were prescribed opioid analgesia at discharge. For those prescribed any opioid on discharge, the median quantity was 100 (i.q.r. 60–200) OMEs. At 7 days, the median quantity consumed was only 40 (7.5–100) OMEs (*P* < 0.001). Complete data on the proportion of prescribed opioids that were consumed by 7 days are available elsewhere^17^. Of note, 197 patients who received a prescription for opioids at discharge (15.0%) did not consume any opioid analgesia in the 24 h before discharge. A total of 2596 patients (60.8%) recalled receiving education about pain management before discharge.

Propensity score matching produced well matched cohorts, demonstrated by the plot of standardized mean differences of included variables (*Fig. S1* and *Table S3*.).

### Postdischarge patient-reported outcomes

**Fig. 2:**
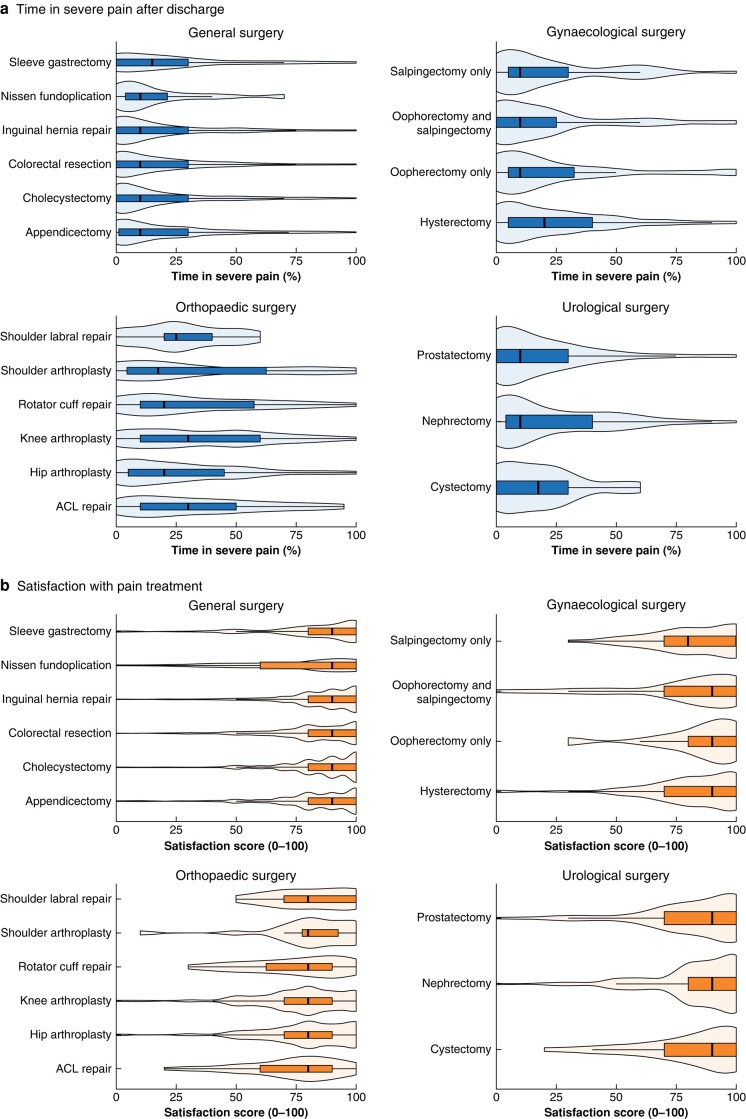
Pain severity after discharge and satisfaction with pain treatment, stratified by procedure **a** Time in severe pain after discharge and **b** satisfaction with pain treatment. Violin and box plots show range, i.q.r., and median values. ACL, anterior cruciate ligament.

The reported time spent in severe pain after discharge, stratified by procedure, is displayed in *Fig. 2a*. After propensity score matching and adjustment for confounding factors in mixed-effects negative binomial regression, patients prescribed opioids spent more time in severe pain after discharge (risk ratio 1.52, 95% c.i. 1.31 to 1.76; *P* < 0.001) (*Tables 3* and *S4*).

Despite the differences in pain severity, there was no difference in patient-reported satisfaction with pain treatment on univariable analysis (median satisfaction rating 90 of 100 in both opioid and no-opioid groups; *P* = 0.157) (*Table 2*), or after propensity score matching and adjustment for confounding factors in mixed-effects linear regression (β coefficient 0.92, 95% c.i. −1.52 to 3.36; *P* = 0.468) (*Tables 3* and *S5*).

**Table 2:** Patient outcomes after discharge from hospital

	No opioids (*n* = 2962)	Opioids (*n* = 1311)	Total (*n* = 4273)	P*
Time in severe pain in week after discharge (%), median (i.q.r.)	10 (0–30)	20 (5–40)	10 (1–30)	< 0.001†
Satisfaction with pain treatment (0–100), median (i.q.r.)	90 (80–100)	90 (80–100)	90 (80–100)	0.157†
**Satisfaction with amount of analgesia provided**				< 0.001
Too little	622 (21.0)	229 (17.5)	851 (19.9)	
Just right	2226 (75.2)	887 (67.7)	3113 (72.9)	
Too much	110 (3.7)	194 (14.8)	304 (7.1)	
Missing	4 (0.1)	1 (0.1)	5 (0.1)	
EQ-5D-5L™ composite score (0–100), median (i.q.r.)	87.9 (76.1–95.0)	80.9 (69.1–89.2)	85.9 (73.8–93.7)	< 0.001†
EQ-VAS score (0–100), median (i.q.r.)	82 (70–90)	75 (60–85)	80 (70–90)	< 0.001†
**Postdischarge medical presentation for pain**				< 0.001
No	2701 (91.2)	1121 (85.5)	3822 (89.4)	
Yes	261 (8.8)	190 (14.5)	451 (10.6)	
**Postdischarge medical presentation for side-effects of medication**				
No	2851 (96.3)	1220 (93.1)	4071 (95.3)	< 0.001
Yes	111 (3.7)	91 (6.9)	202 (4.7)	

Values are *n* (%) unless otherwise indicated. * χ test, except ^†^Kruskal-Wallis test.

**Table 3:** Multivariable analysis of the effect of opioids on outcomes

	Group	Univariable analysis (complete case)		Hierarchical regression analysis (multiple imputation)		Propensity-score matched and regression analysis (multiple imputation)	
		Effect size	*P*	Effect size	*P*	Effect size	*P*
Mixed-effects negative binomial regression*							
Time in severe pain in week after discharge (%)*	No opioids on discharge	Reference		Reference		Reference	
	Opioids on discharge	1.28 (1.15, 1.41)	< 0.001	1.51 (1.34, 1.71)	< 0.001	1.52 (1.31, 1.76)	< 0.001
**Mixed-effects linear regression**†							
Satisfaction with pain treatment (0–100)†	No opioids on discharge	Reference		Reference		Reference	
	Opioids on discharge	0.08 (−0.21, 0.37)	0.586	0.84 (−0.82, 2.51)	0.322	0.92 (−1.52, 3.36)	0.468
EQ-5D-5L™ (composite score, 0–100)†	No opioids on discharge	Reference		Reference		Reference	
	Opioids on discharge	−17.38 (−23.15, −11.61)	< 0.001	−1.48 (−2.99, 0.02)	0.054	−2.27 (−3.82, −0.72)	0.005
EQ-VAS score (0–100)†	No opioids on discharge	Reference		Reference		Reference	
	Opioids on discharge	−4.24 (−5.09, −3.40)	< 0.001	−1.64 (−3.37, 0.09)	0.064	−2.82 (−4.51, −1.14)	0.002
**Mixed-effects logistic regression**‡							
Postdischarge medical presentation for pain‡	No opioids on discharge	Reference		Reference		Reference	
	Opioids on discharge	1.73 (1.37, 2.17)	< 0.001	1.32 (0.97, 1.80)	0.080	1.01 (0.70, 1.46)	0.948
Postdischarge medical presentation for medication side-effects‡	No opioids on discharge	Reference		Reference		Reference	
	Opioids on discharge	1.83 (1.31, 2.55)	< 0.001	2.92 (1.84, 4.63)	< 0.001	2.38 (1.36, 4.17)	0.004

Values in parentheses are 95% confidence intervals. Effect sizes are shown as *risk ratios, †β coefficients, and ‡ORs.

**Fig. 3:**
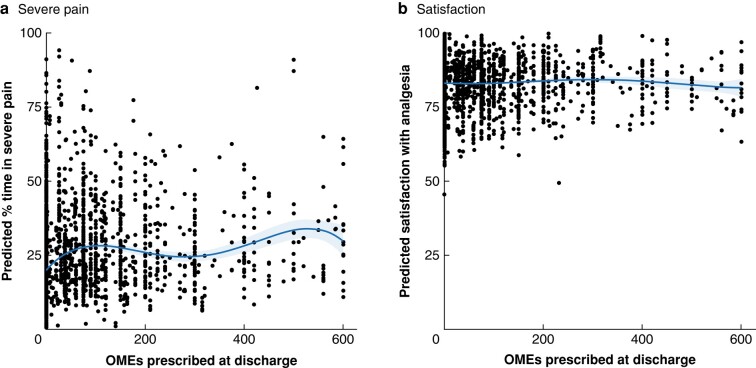
Relationship between quantity of oral morphine equivalents prescribed at discharge and modelled time in severe pain and modelled patient satisfaction with pain treatment **a** Modelled time in severe pain and **b** modelled patient satisfaction with pain treatment adjusted for patient demographics, co-morbidity, operation type, duration and indication, preoperative opioid and non-opioid analgesia, postoperative complications, and oral morphine equivalent (OME) requirements 24 h before discharge. Each individual dot represents an individual patient. The solid lines and shaded areas represent the polynomial regression lines and 95% confidence intervals respectively.

There was no dose-dependent relationship between the quantity of opioids prescribed on discharge and pain severity or patient-reported satisfaction in both unadjusted (*Fig. S2*) and adjusted analyses (*Fig. 3*).

### Quality of life and re-presentation to healthcare

Before risk adjustment, patients prescribed opioids reported poorer quality of life at 7 days after discharge compared with those not prescribed opiates when measured by a composite EQ-5D-5L™ score (median 80.9 *versus* 87.9; *P* < 0.001) and EQ-VAS score (75 *versus* 82; *P* < 0.001). This association persisted after adjustment in mixed-effects linear regression and with propensity score matching; quality of life was poorer as measured by both EQ-5D-5L™ score (β coefficient −2.27; *P* = 0.005) (*Tables 3* and *S6*) and EQ-VAS score (β coefficient −2.82; *P* = 0.002) (*Tables 3* and *S7*). Opioid prescription at discharge did not increase the likelihood of patients seeking additional healthcare for pain relief (OR 1.01, 95% c.i. 0.70 to 1.46; *P* = 0.948) (*Tables 3* and *S8*), but increased the risk of presentation to healthcare owing to side-effects of medication (OR 2.38, 1.36 to 4.17; *P* = 0.004) (*Tables 3* and *S9*).

### Excess and insufficient analgesia prescription

Overall, 14.8% of patients prescribed opioid analgesia felt that they were prescribed too much pain relief medication, compared with 3.7% of those prescribed only non-opioid analgesia. Conversely, 17.5% of patients prescribed opioids felt they were prescribed too little pain relief medication, compared with 21.0% of those prescribed no opioids (*P* < 0.001) (*Table 2*).

Factors associated with reporting receiving too little pain relief on multivariable analysis included female sex (OR 1.25, 95% c.i. 1.03 to 1.51; *P* < 0.001), preoperative regular use of opioid analgesia (OR 1.94, 1.32 to 2.82; *P* < 0.001), ASA grade IV–V (OR 2.66, 1.27 to 5.54, *versus* ASA I), postoperative complications (OR 1.41, 1.13 to 1.76, for Clavien–Dindo grade I–II *versus* no complications; *P* < 0.003), and specific orthopaedic or urological procedures (*Table S10*).

Patients who underwent orthopaedic procedures reported severe pain more frequently in the 7 days after discharge than those who had abdominal procedures (median 30 (i.q.r. 10–50) and 10 (0–30)% respectively; *P* < 0.001) (*Fig. 2a*), and reported a lower level of satisfaction with pain relief after discharge (median 80 (i.q.r. 70–90) *versus* 90 (80–100) of 100; *P* < 0.001) (*Fig. 2b*). Orthopaedic patients reported lower quality-of-life scores after discharge than those who had abdominal procedures (median EQ-VAS score 70 (i.q.r. 60–80) *versus* 80 (70–90) of 100; *P* < 0.001), and were more likely to seek further analgesia (18.3 *versus* 9.3%; *P* < 0.001), and re-present to healthcare owing to side-effects of analgesia (9.1 *versus* 4.0%; *P* < 0.001).

### Geographical variation

The 1923 patients from HICs as defined by the OECD were prescribed more opioids on average than the 2350 patients from LMICs (median 37.5 (0–112.5) and 0 (0–0) OMEs respectively; *P* < 0.001). Patients from HICs had nine times higher odds of receiving opioids on surgical discharge than those from LMICs after adjusting for case mix, patient co-morbidity, postoperative complications, and analgesic needs after propensity score matching (adjusted OR 9.10, 95% c.i. 7.70 to 11.10) (*Table S11*). Similar results were found without propensity score matching (adjusted OR 10.0, 8.30 to 12.50).

Although there was a statistically significant difference, there were no clinically significant differences in time spent in severe pain (median 10 (i.q.r. 0–30)% in first week in HICs *versus* 10 (3–30)% in LMICs; *P* < 0.001) or patient satisfaction between HICs and LMICs (median 90 (80–100) and 85 (70–100) respectively; *P* < 0.001).

Geographical differences in patients and outcomes between Asia Pacific, North America, Central and Latin America, Middle East and North Africa, Europe and Central Asia, Sub-Saharan Africa, and South Asia are presented in full in *Tables S12* and *S13*.

## Discussion

This multinational prospective cohort study demonstrated that prescription of opioids at hospital discharge after common surgical procedures was not associated with improved treatment satisfaction compared with opioid-free analgesia. Furthermore, increasing opioid prescription quantities was not associated with changes in pain severity or patient satisfaction. Opioid prescription was associated with increased presentation for management of pain medication side-effects, without an associated reduction in presentations for further pain management. This study expands on previous work limited to mostly minor elective day-case procedures^11,25–27^, by including acute operations, major visceral resections, and major orthopaedic procedures. The present study provides prospective, international data to inform discharge analgesia prescription after common surgical procedures, and highlights that opioid-free analgesia at discharge can be the default rather than the exception.

It is becoming increasingly apparent across a range of surgical procedures that most patients do not benefit from opioid pain relief at discharge, with only a small targeted set of patients requiring opioid prescriptions on discharge^11,25,28^. In this study, female sex, opioid use before surgery and on discharge, lower limb orthopaedic surgery, elective procedures, and mild postoperative complications (Clavien Dindo grades I–II) were associated with increased time spent in severe pain, consistent with findings reported in other reviews. Clinician concern surrounding patient dissatisfaction after discharge and healthcare reutilization owing to uncontrolled pain is a major driver of opioid overprescription^7,29^, but this was not shown in the present cohort and others^23,30^. Similar studies conducted in general surgical procedures^7,10^, breast procedures^26,27^, major abdominal and urological procedures^11,28^, gynaecological procedures^31^, as well as orthopaedic sports operations^32^ have shown that decreasing opioid prescriptions or opioid-free analgesia following discharge does not decrease patient satisfaction scores after surgery. On the contrary, healthcare utilization for patients experiencing side-effects of medication was increased for those with opioid analgesia compared with those receiving opioid-free analgesia, a finding that replicates previous studies^11^. The present study therefore reinforces previous findings that increasing opioid prescription on discharge is not independently associated with pain severity, and indicates that clinicians should not prescribe opioids as a panacea for postoperative pain.

Marked global variation in opioid prescribing was demonstrated in this cohort. Similar geographical differences have been demonstrated in other studies, with the USA and Canada typically seeing significantly higher quantities of opioids being prescribed for the same procedures compared with other countries including Sweden, China, Lebanon, Brazil, Mexico and the Netherlands^33,34^. This study extends the findings of previous studies and demonstrates that, at follow-up 7 days after discharge, there is no clinically meaningful difference in reported pain levels when the patient cohort is stratified by geography. Current geographical variations in opioid prescribing likely reflect entrenched medicocultural practices rather than evidence-based pain management.

Patient outcomes differ across different surgical procedures, with patients who undergo orthopaedic procedures experiencing greater postoperative pain and lower satisfaction than those having abdominal procedures. Given that many patients undergoing arthroplasty are regular users of opioids in the preoperative phase, those in this situation are at a higher risk of uncontrolled postoperative pain and chronic opioid use after surgery^35^. However, regardless of procedure, a multimodal approach to pain management is required, with preoperative assessment for high-risk pain characteristics, appropriate modulation of patient expectations, multimodal analgesia, and pain treatment planning on discharge^36^. Preoperative counselling on pain management and opioid use may reduce patient-reported pain scores, increase the likelihood of patients using non-pharmacological therapies, and increase levels of function at 6 months after operation^37^.

For this study, it was possible to collect prospective international patient-reported data across a range of surgical specialties with high rates of follow-up. Nevertheless, there are several limitations. After extensive co-variate adjustment, matching, and subgroup analysis, this study showed results consistent with randomized data^11^. However, observational data are not a substitute for randomized data for deducing causal relationships, and the results need to be interpreted with this in mind. Furthermore, although extensive physical co-morbidity data were collected, it was not possible to adjust for co-existing anxiety, depression or pain catastrophizing, which are other factors associated with pain severity^23^. At follow-up, patient-reported outcomes may be prone to recall bias, but this was minimized by the relatively short duration of follow-up. Although a 7-day follow-up may be considered a short time frame for outcomes assessment, this was selected as clinical care standards recommend limiting the duration of usual discharge opioid prescriptions to less than 7 days^38,39^. Lastly, cultural differences in pain perception and reporting may confound patient recall of outcomes^40^. This potential risk to the results was mitigated by factoring in centre and country-level effects into mutivariable models.

The present results suggest that opioid prescribing at surgical discharge is not associated with reduced patient satisfaction, but with an increased risk of presentation to healthcare owing to the side-effects of pain medication. Increasing the quantity of prescribed opioid was not associated with changes in patient-reported pain. Further studies should focus on developing prescribing guidelines for high-risk patients, including those with preoperative opioid needs, and for specific procedures associated with high analgesia requirements.

## Supplementary Material

znad421_Supplementary_Data
