# Corrigendum to: Impact of opioid-free analgesia on pain severity and patient satisfaction after discharge from surgery: multispecialty, prospective cohort study in 25 countries

**DOI:** 10.1093/bjs/znae152

**Published:** 2024-06-25

This is a corrigendum to: TASMAN Collaborative, Impact of opioid-free analgesia on pain severity and patient satisfaction after discharge from surgery: multispecialty, prospective cohort study in 25 countries, *British Journal of Surgery*, Volume 111, Issue 1, January 2024, znad421, https://doi.org/10.1093/bjs/znad421

In the originally published version of this manuscript three authors were inadvertently omitted from the list of authors provided in the supplementary materials; Jason Chung, Sewni Samarawickrama, and Flavio Ordones.

This error has been corrected in the supplementary materials online.

